# The Functional Signature of Decision Making Across Dyads During a Persuasive Scenario: Hemodynamic fNIRS Coherence Measure

**DOI:** 10.3390/s25061880

**Published:** 2025-03-18

**Authors:** Michela Balconi, Roberta A. Allegretta, Carlotta Acconito, Federica Saquella, Laura Angioletti

**Affiliations:** 1 International Research Center for Cognitive Applied Neuroscience (IrcCAN), Università Cattolica del Sacro Cuore, Largo Gemelli 1, 20123 Milan, Italy; michela.balconi@unicatt.it (M.B.); carlotta.acconito1@unicatt.it (C.A.); laura.angioletti1@unicatt.it (L.A.); 2 Research Unit in Affective and Social Neuroscience, Department of Psychology, Università Cattolica del Sacro Cuore, Largo Gemelli 1, 20123 Milan, Italy; 3 Faculty of Medicine, Università degli Studi di Milano, via Festa del Perdono, 7, 20122 Milan, Italy; federica.saquella@studenti.unimi.it

**Keywords:** persuasion, shared decision making, fNIRS, hyperscanning, hemodynamic coherence

## Abstract

Introduction: Within a shared decision-making process, persuasion dynamics develop as a communication sub-process that can be characterized by different phases. This study examines hemodynamic functional Near-Infrared Spectroscopy (fNIRS) coherence measures in dyads of decision-makers. The interaction occurs in two phases: Phase 1, where the persuader (Pr) introduces the decision topic and uses persuasive strategies, and Phase 2, where the Persuaded (Pd) responds and may agree with the Pr’s selected option. Method: Fourteen dyads participated, with fNIRS measuring oxygenated (O_2_Hb) and deoxygenated (HHb) hemoglobin concentration changes in the prefrontal cortex (PFC) during both phases. Hemodynamic coherence within dyads was explored through the computation of a dissimilarity index (Euclidean distance). Results: Phase 2 showed increased HHb dissimilarity, indicating greater divergence in brain activity during the Pd’s response phase. Discussion: These findings suggest that, during persuasion, when Pd responds, there is increased dissimilarity in cognitive and neural processes, without implying a loss of synergy. The study highlights the importance of interactional dynamics in shaping decision outcomes and underscores the potential of fNIRS as a non-invasive tool for monitoring brain activity in clinical and collaborative settings.

## 1. Introduction

Decision making is defined as a cognitive and emotional process enabling an individual to choose a specific course of action from numerous options and possibilities [1,2]. However, in different contexts and across various fields, such as business, politics, organizational management, social interactions, and clinical settings, decision making is not only an individual matter (in which a single person makes a decision for him/herself), but also a shared and collective process in which two or more individuals collaborate to discuss, evaluate, and select, from a range of alternatives, the most suitable for each involved party [3].

This shared approach emphasizes dialogue, collaboration, and, in some cases, persuasion among individuals to support decision making [4]. Indeed, the act of deliberately engaging with someone to influence their attitudes or behavior can be understood as a form of social influence, defined as persuasion [5]. Persuasion involves a deliberate attempt or intention by an individual, known as the “persuader” (Pr), to influence the beliefs, attitudes, or actions of the target, referred to as the “persuaded” (Pd) [5].

Historically, research on persuasion has predominantly focused on attitudes and behaviors, and references have been made to a process with distinct phases involving cognitive and attitudinal changes. Notably, Aquino et al. [6] noted that spreading information—such as ideas, viewpoints, and behaviors—depends on two fundamental mechanisms: initially, upon first exposure, individuals start to unconsciously reflect on the message’s content; then, in the evaluation stage, they determine the message’s personal significance and consciously develop an attitude in response to it.

In real-world scenarios, persuasion often occurs in group conversations involving complex interactions between the message creator and the audience. Interestingly, in recent years, neuroscientists have increased our comprehension of persuasion, focusing particularly on the underlying mechanisms that traditional self-report methods may not effectively capture [7]. However, while extensive research has explored the neural basis of producing persuasive messages, the interpersonal brain areas that convert these messages into influence on individuals or groups remain poorly understood. This gap limits our understanding of the brain systems involved in persuasion during naturalistic interactions implying decision making [8].

### 1.1. Neuroimaging Studies of Persuasion in Shared Decision Making

In the past ten years, an increasing number of studies utilizing functional magnetic resonance imaging (fMRI) have focused on the topic of persuasion [5], revealing key brain structures involved in the persuasion process both in the Pr and in the Pd.

Notably, studies reveal how information becomes deeply embedded in the brain of the Pd [9], emphasizing the significant role played by the value system, which includes the ventromedial prefrontal cortex (vPFC), the medial prefrontal cortex (mPFC), and the ventral striatum [5,10,11]. The value system is thought to reflect the integration of a message’s significance into the Pd’s self-concept, serving as a key predictor of whether persuasive messages succeed in influencing behavior. Nonetheless, the majority of traditional neuroimaging studies on persuasion use fMRI and often focus on individuals, missing the dynamic interactions between the Pr and the Pd in a shared decision-making process, overlooking the dynamic and reciprocal interactions between the Pr and the Pd from an interpersonal neuroscience perspective [12]. Effective communication leads the Pd to align their attitudes and behaviors with the message source [11], while shared brain activity fosters cognitive, emotional, and behavioral synchronization [13].

Functional near-infrared spectroscopy (fNIRS) has gained attention as an alternative neuroimaging method with notable advantages over fMRI, including enhancing ecology in neuroscience research [14,15]. This technique assesses brain activity by tracking the propagation of infrared light through the cortex: indeed, when specific brain regions are activated, neurons require more oxygen, leading to an increase in regional metabolism. This heightened oxygen demand triggers an elevated regional cerebral blood flow (rCBF). Studies have shown that the increase in rCBF surpasses the actual oxygen needs of the tissue, resulting in higher levels of oxygenated hemoglobin (O_2_Hb) and lower levels of deoxygenated hemoglobin (HHb), both of which serve as markers of neural activation [16,17,18,19]. Both signals are of great interest to consider and further explore in relation to activation or inhibition mechanisms. Consequently, through the use of near-infrared light and optical sensors, fNIRS can be regarded as a valuable method for studying neural activity in less restrictive and more realistic environments [20].

This advanced and efficient technology is particularly well-suited for assessing and tracking brain function, especially in clinical environments, not as a diagnostic tool but rather as an assistive device that monitors functional brain activity and provides indirect insights into potential abnormalities in brain functionality. Indeed, in conditions such as Alzheimer’s, schizophrenia, dyslexia, addiction, ADHD, epilepsy, and depression, fNIRS serves as a predictive modality by distinguishing functional hemodynamic activity in conjunction with behavioral testing, aiding in the understanding of these conditions [21].

To better understand the complexity of synchronization and interpersonal dynamics, the “hyperscanning” paradigm in neuroscience has facilitated a shift from single-subject studies to a “two-person neuroscience” approach [22], enabling the simultaneous recording of brain activity from two or more interacting participants [23], generating spatiotemporal maps of the brain regions involved in social interactions [24,25,26].

To date, to the best of our knowledge, only a study has explored interpersonal neural synchronization between a Pr and a Pd during a task that relies on a hypothetical scenario (the Arctic Survival Task) [12] that, however, is distant from everyday social interactions.

Specifically, this study explores how neural coupling between a Pr and a Pd supports effective persuasion and predicts outcomes in naturalistic settings and highlights that the neural coupling of O_2_Hb serves as a critical indicator of persuasion success, specifically in predicting the compliance of the Pd. Results showed that persuasive arguments elicited stronger neural coupling in key brain regions [superior temporal gyrus (STG), superior frontal gyrus (SFG), and inferior frontal gyrus (IFG)] compared to non-persuasive arguments. Analyses revealed that neural synchronization occurs early during persuasion, marking critical moments when the receiver aligns with the persuader’s arguments. Another study [27] analyzed brain activity associated with face-to-face conversations between two individuals through fNIRS with a hyperscanning paradigm. Specifically, the authors found that the frontoparietal system, which includes the bilateral dorsolateral prefrontal cortex (dlPFC), left supramarginal gyrus, angular gyrus, and superior temporal gyrus, exhibited heightened activity during conversations in the context of disagreement. On the other hand, conversations during agreement were marked by increased activity in a social and attention network, which involves the right supramarginal gyrus, bilateral frontal eye fields, and left frontopolar regions. Additionally, these social and visual attention networks showed greater synchrony across the brain during agreement compared to disagreement.

Furthermore, a recent study by Angioletti et al. [28] investigates hemodynamic and autonomic markers of persuasion, highlighting increased O_2_Hb dissimilarity when the persuaded responds. Moreover, results showed that HR dissimilarity increased as the persuaded spoke, indicating disrupted synergy, while the HRV dissimilarity was higher when the persuader spoke, suggesting stress regulation differences. However, compared to the current study, this recent study did not take into consideration the efficacy of the persuasion process, the lateralization of brain activity, and the role of individual differences. Despite the interesting findings, these studies mainly focus on the exploration and interpretation of O_2_Hb, but it would be of particular interest to focus also on the exploration and interpretation of Hbb.

Most studies highlight the importance of the PFC in the persuasive process [5,10,11,12]. In addition to this, recent studies have examined the lateralization of the PFC, suggesting that the right and left hemispheres may play distinct roles in the cognitive and emotional aspects of persuasion, already emphasized by extensive literature: the specialization of the right hemisphere in processing affective messages and the specialization of the left hemisphere in processing cognitive messages [29].

Notably, Aquino et al. [6] further corroborated the lateralization hypothesis by directly comparing affective and cognitive persuasive information, illustrating that hemisphere specialization is evident both during the reading of affective versus cognitive messages and in the evaluation of objects previously described by these messages. These findings are strictly related to individual differences, including the so-called structural matching effect [30] according to which persuasion can appeal to either affective aspects (e.g., the emotions evoked by a message or a product) or cognitive aspects (e.g., factual attributes of a message or a product). Research suggests that these messages are more effective in driving attitude changes when their content aligns with the Pd’s affective or cognitive attitude [31].

### 1.2. The Relation Between Brain Activity and Individual Differences

Among individual differences, personality traits [assessed through the 10-item Big Five Inventory (BFI) [32]] hold a key role in the effectiveness of persuasive communication [33,34]. For instance, research has demonstrated that individuals who are agreeable are more likely to be persuaded by those they find likeable [35], whereas conscientious individuals are more inclined to be influenced by authoritative figures.

However, in the context of shared decision making, it might be plausible that other individual differences play an important role in the persuasive process, including decision-making styles and maximization tendencies. Notably, the decision-making style [which is usually investigated through the General Decision-Making Style (GDMS) [36,37]] refers to the consistent patterns of behavior an individual displays in situations requiring a decision [37]. It reflects a habitual approach to decision making rather than a fixed personality trait, highlighting the dynamic nature of how individuals respond to decision-making scenarios.

Instead, Schwartz [38] observed that, in the decision-making process, individuals might adopt one of two tendencies: being a “satisficer”, who is satisfied with a solution that is adequate or “good enough”, or a “maximizer”, who strives to find the optimal choice. These tendencies—which are usually assessed via the Maximization Scale (MS) [39,40]—not only influence the outcomes of their decisions, but also have significant implications for their psychological well-being [38,41].

Furthermore, since persuasion plays a critical role in negotiation, as it facilitates the necessary movement or shifting of interests between two parties [42], negotiation skills as well might play a key role in the success of the persuasive process. To investigate and assess such skills, a useful tool is the Negotiations Self-Assessment Inventory (NSAI) [43].

Interestingly, different studies have confirmed the importance of individual differences in decision-making behavior (particularly in group decision-making dynamics) from a neural perspective [44]. Notably, Lepine and Van Dyne [45] identified a connection between cooperative behavior and certain Big Five personality traits, including conscientiousness, extroversion, and agreeableness. Similarly, Zhang et al. [44] found that agreeableness appears to serve as a key predictor of interbrain synchrony during group decision making. Furthermore, authors found that the agreeableness trait and defection decisions were negatively correlated in the dorsolateral prefrontal cortex (dlPFC), which primarily facilitates executive functions, such as decision making. Therefore, individuals with higher levels of agreeableness may prioritize shared goals and cooperation within group settings. This tendency reduces the likelihood of engaging in defective behavior (i.e., decisions that prioritize individual gain over group benefits) [44].

Given these premises, it would be of particular interest to further investigate the role of individual differences in shared decision making from a neural perspective by taking into account not only personality traits, but also decision-making style, maximization tendencies, and negotiation skills.

## 2. Materials and Methods

### 2.1. Sample

A total of 28 university students (8 males: M_age_ = 26.25, SD = 6.76; 20 females: M_age_ = 25.15, SD = 5.8), recruited through a non-probabilistic convenience sampling method, took part in the study (see Table 1). A priori power analysis, conducted using G*Power version 3.1.9.7, determined that a minimum of 21 participants was sufficient for repeated-measures ANOVA with four measurements and three groups (effect size f = 0.40, α = 0.05, power = 0.95). Therefore, the final sample size of N = 28 was deemed adequate for testing the study hypotheses.

Participants were randomly assigned to be the Pr or the Pd and then paired to form 14 dyads, each consisting of two individuals of the same gender who had no prior acquaintance or familiarity before the experimental session. Participation was entirely voluntary, with no compensation provided. All participants were right-handed and had normal or corrected-to-normal vision. The following exclusion criteria were applied during recruitment: a history of psychiatric or neurological disorders, significant levels of depression, impaired global cognitive functioning, abnormal short- or long-term memory, or ongoing treatment with psychoactive drugs that could affect cognitive performance.

The study was approved by the Ethics Committee of the of the Catholic University of the Sacred Hearth, Milan (approval code: 125/24—Valutare il Decision Making: consapevolezza e metacognizione decisionale; approval date: 23 July 2024) and conducted in accordance with the Declaration of Helsinki (2013) and the GDPR—Reg. UE 2016/679 and its ethical guidelines.

### 2.2. Experimental Procedure

The experimental procedure was conducted in a quiet, moderately darkened room, where participants were seated on comfortable chairs positioned to facilitate face-to-face interaction and provided informed consent before the beginning of the experiment. The experimental procedure consisted of three main steps:

(i) Persuasive interaction, in which participants engaged in a face-to-face persuasive interaction during which fNIRS data were recorded continuously from both the Pr and the Pd;
(ii) Perception of the persuasive interaction, where, following the interaction, participants evaluated their experience and perception of the persuasive process through post-task items derived and modified from the Group Questionnaire [46]
(iii) Assessment of individual differences, in which participants were asked to fulfil the GDMS [36,37], 10-item BFI [32], MS [39,40], and NSAI [43].

**Figure 1:**
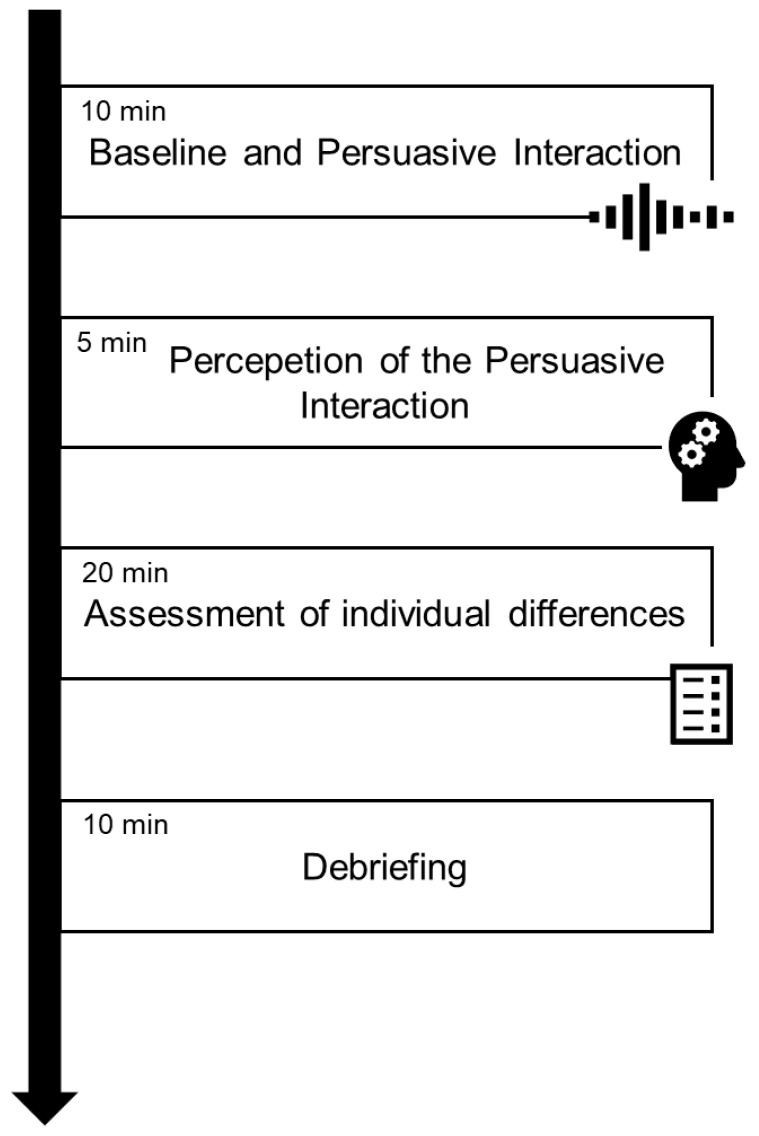
Experimental procedure.

Before starting the interaction, a 120 s eyes-open fNIRS resting-state baseline was recorded for each participant. The whole procedure lasted approximately 45 min (see Figure 1).

#### Persuasive Interaction

The persuasive process was divided into two phases involving firstly the Pr and, subsequently, the Pd. The interaction was structured as a realistic verbal exchange centered on a specific topic, that is, group decision making. In this exchange, one participant assumed the role of the Pr, aiming to persuade the Pd, while the Pd decided whether to accept and endorse the Pr’s proposed approach. Each participant received distinct instructions before the interaction.

Specifically, prior to Phase 1 of the persuasion process, the Pr was thoroughly briefed about his/her role. They were informed that the discussion would focus on a hypothetical, everyday scenario—a member of a workgroup failing to align with the group’s values and practice—by giving the following instructions:

*“A member of a workgroup does not appear to be in line with the ideals and style of the group. Think about your ideal workgroup. Choose the sentence that most closely aligns with the decision and behavior you would expect from your group.”*

The Pr’s responsibility was to select a statement from eight provided options that best addressed the situation: (i) consulting external experts outside the group and company, with decisions based on their recommendations; (ii) adopting the opinion of the most innovative group member; (iii) following the perspective of the group’s most experienced member; (iv) seeking advice from colleagues outside the team and basing decisions on their input; (v) allowing all group members to voice their opinions, reaching a consensus on the issue and making a collective decision; (vi) adopting the decision supported by the majority of the group; (vii) splitting the group when disagreements persist, with each faction pursuing its direction, while I attend the meeting and try my best; (viii) consulting reliable sources and making a decision based on their information. Then, they received the following instructions to engage in the persuasive interaction within the decision-making scenario:

*“Now, present this scenario to the other participant and attempt to persuade them that your chosen approach for acting within an ideal group is the most effective and should be adopted by both of you in a group setting.”*

Therefore, during Phase 1 of the interaction, each Pr introduced the scenario as outlined in the experimental instructions, employing persuasive strategies to establish and articulate their goal. In this stage, the Pd listened to the Pr, who took the lead as the speaker, steering the conversation and executing their strategies.

In Phase 2, the focus shifted primarily to the responses of the Pd, who took on the role of the speaker while the Pr listened attentively to their responses. Phase 1 was the same for all Pr, while Phase 2 was the same for all Pd. Overall, the persuasive interaction lasted for approximately three minutes.

### 2.3. Perception of the Persuasive Interaction

At the end of the persuasive interaction, the effectiveness of the persuasion process within each dyad was assessed using a Likert-scale item completed by both the Pr and the Pd which stated: “*The idea of the group I had before the interaction has changed, in accordance with the other member’s idea*” and they were asked to respond on a five-point Likert scale, where 1 indicated “completely agree” and 5 indicated “completely disagree”. Based on the participants’ responses, the average score for the sample was calculated, and the dyads were categorized according to the perceived efficacy of the persuasive interaction. Specifically, three categories emerged. (i) Effective dyads: both members rated the persuasive interaction as effective (N = 5 dyads); (ii) ineffective dyads: both members rated the interaction as ineffective (N = 6 dyads); (iii) mixed dyads: one member rated the interaction as effective, while the other rated it as ineffective (N = 3 dyads).

#### Assessment of Individual Differences

At the beginning of the experimental procedure, participants completed four self-report questionnaires: the 10-item BFI [32], the GDMS [36,37], the MS [39,40], and the NSAI [43].

The BFI-10 is a tool designed to measure the Big Five personality dimensions using ten items. It evaluates five key personality traits: extraversion (characterized by enthusiasm and extroversion), agreeableness (reflects likeability and warmth in interactions), conscientiousness (demonstrates traits of organization and self-discipline), emotional stability (indicates calmness, resilience, and emotional balance), and openness (captures imagination, curiosity, and receptiveness to new experiences).

The GDMS questionnaire uses a five-point Likert scale to classify decision-making approaches into five distinct styles: rational (marked by a comprehensive and systematic search for information, coupled with logical evaluation of alternatives), intuitive (characterized by a focus on overarching aspects and a reliance on feelings or instincts), dependent (reflects a preference for seeking advice and guidance from others, indicating a need for external support in decision making), avoidant (involves a tendency to delay or avoid making decisions altogether), and spontaneous (represents individuals who make quick and impulsive decisions without prolonged deliberation).

The MS uses a seven-point Likert scale to assess individual differences in the tendency to maximize choices across three dimensions: alternative search (investing significant time and resources in exploring all possible options), decision difficulty (experiencing challenges in making decisions when faced with numerous options), and high standards (maintaining elevated expectations for oneself and the outcomes one pursues).

The NSAI uses a five-point Likert scale to assess individual tendencies to utilize five distinct negotiation styles: avoidance (individuals who avoid confrontation ignoring the issues at hand and the relational dynamics involved), aggression (aggressive negotiators prioritize their own goals, seeking to win regardless of the impact on others), accommodation (prioritizing relational dynamics, often at the expense of the individual’s own interests and needs), compromise (aiming to find a balanced solution by seeking middle ground), and collaboration (focusing on mutually beneficial outcomes and being sensitive to relationship dynamics). The participants were asked to rate how often they agreed with 25 statements related to these negotiation styles using a Likert scale (ranging from 0 to 5: never, rarely, sometimes, occasionally, frequently, always).

### 2.4. fNIRS Data Acquisition and Processing

**Figure 2:**
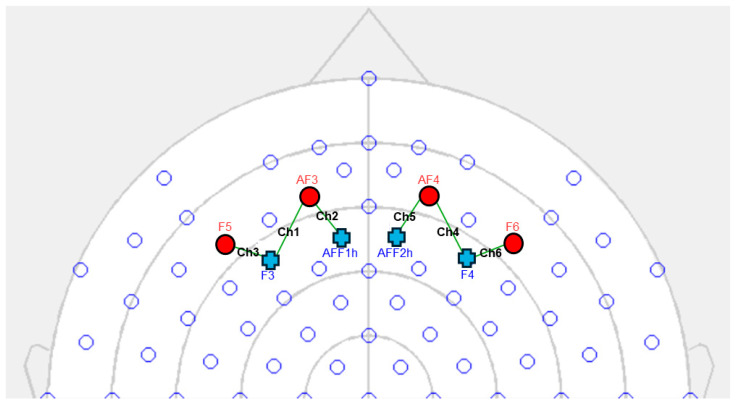
fNIRS setup. The four light sources (AF3, AF4, F5, F6) are marked with a red circle, whereas the four detectors (AFF1h, AFF2h, F3, and F4) are marked with a blue cross. The six established measurement channels are marked in black: Ch1 (AF3-F3), Ch2 (AF3-AFF1h), Ch3 (F5-F3), Ch4 (AF4-F4), Ch5 (AF4-AFF2h), and Ch6 (F6-F4).

Using a six-channel optodes matrix from the NIRScout System (NIRx Medical Technologies, LLC, Los Angeles, CA, USA), the variations in the concentrations of oxygenated hemoglobin (O_2_Hb) and deoxygenated hemoglobin (HHb) were measured. Four light sources/emitters and four detectors were positioned on the scalp using an fNIRS cap, following the 10/5 international standard [47]. The emitter–detector distance between consecutive optodes was set to 30 mm, utilizing two wavelengths of near-infrared light (760 and 850 nm). A probabilistic atlas available in the fOLD software (fNIRS Optodes’ Location Decider, version 2.2.1) [48] was used for the positioning of the sources, detectors, and spacing between them, ensuring alignment with the underlying functional regions and the most relevant Brodmann areas [49,50]. The analysis concentrated on the frontal brain regions, particularly the prefrontal cortex (PFC) to explore its activity during the persuasion process (Figure 2).

A sample rate of 6.25 Hz was used to collect the signals recorded from the six channels via the NIRStar Acquisition Software version 12.4 (NIRx Medical Technologies LLC, 15 Cherry Lane, Glen Head, NY, USA). Subsequently, it was processed and converted using the nirsLAB software (v2014.05; NIRx Medical Technologies LLC, 15 Cherry Lane, Glen Head, NY, USA), generating mmol·mm values that represent fluctuations in O_2_Hb and HHb concentrations for each channel. During pre-processing, to refine the data, a digital band-pass filter (0.01–0.3 Hz) was applied to the raw O_2_Hb and HHb signals [51,52].

**Figure 3:**
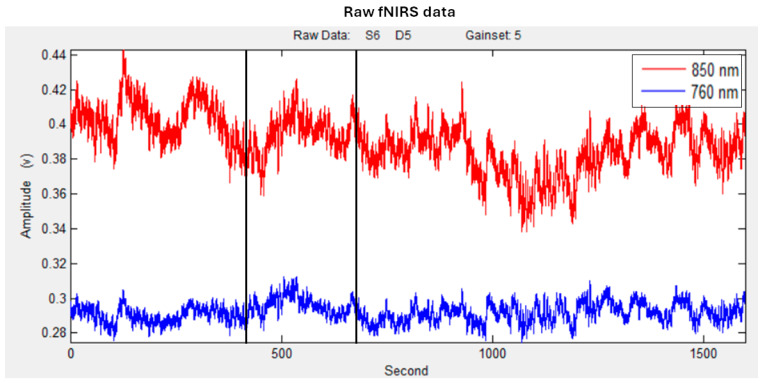
Example of raw fNIRS O_2_Hb (in red) and Hbb (in blue) time series. Black lines represent the 180 s time window of the persuasion process.

The raw time-series data (see Figure 3) were visually inspected subject by subject during both the experimental phase and signal processing to detect noisy channels caused by motion artifacts or amplitude fluctuations. Channels with poor optical coupling or lacking ~1 Hz heartbeat oscillations were excluded from the analysis [53]. A linear-phase Finite Impulse Response (FIR) filter (0.3 Hz) was applied to account for respiration, ensuring a symmetric impulse response [54,55].

After pre-processing the biosignals, the mean concentration for each channel across the phases was calculated. The effect size for each condition was determined by calculating the mean concentration difference between baseline and trial conditions for each channel and subject. Effect sizes (Cohen’s d) were calculated as follows: D = (m1 − m2)/s, where m1 and m2 are the mean concentration levels for the baseline and trial, respectively, and s is the baseline SD. The effect sizes from the six channels were averaged to enhance the signal-to-noise ratio. Although the raw fNIRS data were initially relative values, the normalized effect sizes were averaged, as they are unaffected by the differential pathlength factor (DPF). For statistical analysis, the channels were grouped and averaged to calculate the lateralization factor for the left (Ch1-Ch2-Ch3) and right (Ch4-Ch5-Ch6) hemispheres, corresponding to the left and right prefrontal cortex (PFC).

### 2.5. Data Analysis

#### 2.5.1. fNIRS Interbrain Data Analysis

The study examined interbrain dynamics by assuming that the roles of Pr and Pd during interactions could be differentiated based on distinct hemodynamic activation patterns within the two different phases. To quantify these differences, Ward’s method of Euclidean distance (Eu_dis_) between Region of Interest (ROI) (left and right) and Phase for O_2_Hb and HHb within each dyad was used, serving as a dissimilarity index.

Subsequently, two repeated measures ANOVAs were conducted with ROI (two levels: left, right) and Phase (two levels: Phase 1, Phase 2) treated as within-subject factors and Efficacy (three levels: effective, ineffective, mixed) included as a between-subject factor. The dissimilarity index for O_2_Hb and HHb was used as the dependent variable in each analysis.

For all repeated measures ANOVAs, Greenhouse-Geisser corrections were applied to adjust the degrees of freedom when necessary. Significant interactions were further explored through pairwise comparisons, with the Bonferroni correction applied to control for biases due to multiple comparisons. Effect sizes for significant results were measured using eta squared (*η*^2^), with statistical significance set to α = 0.05. The normality of the data was confirmed in a preliminary analysis phase through skewness and kurtosis tests.

Through a preliminary analysis, potential gender-related biases were assessed and ruled out. No statistically significant main or interaction effects involving gender were found; therefore, this variable was not included in the analyses.

#### 2.5.2. Correlation Analysis Between fNIRS and Psychometric Data

To investigate whether individual differences (i.e., decision-making styles, maximization tendency, personality trait, and negotiation skills) were linked to a ROI of the hemodynamic activity during both phases, Pearson’s correlations were conducted between (i) Eu_dis_ of O_2_Hb in the left and right hemispheres during Phase 1 and Phase 2 with Eu_dis_ of GDMS, MS, 10-item BFI, NSAI and (ii) Eu_dis_ of HHb in the left and right hemispheres during Phase 1 and Phase 2 with Eu_dis_ of GDMS, MS, 10-item BFI, NSAI.

## 3. Results

**Figure 4:**
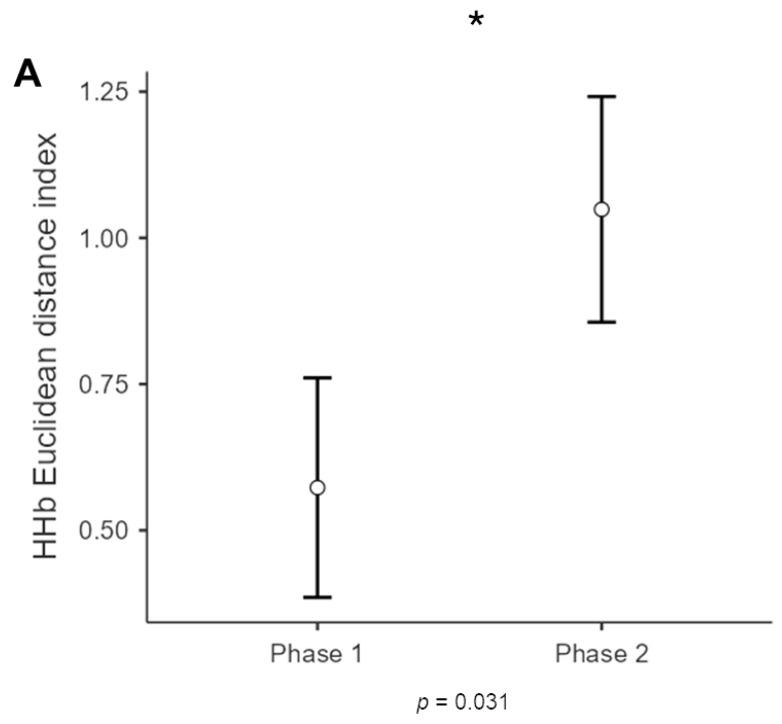
HHb results of dyads. The bar graph shows a significant difference for HHb in the two phases of the interaction, for which Phase 2 showed higher dissimilarity (Eu_dis_) compared to Phase 1 at the interbrain level. The bars represent ± 1 standard error and the star (*) marks statistically significant effects.

For HHb, a significant main effect for phase [F(1,11) = 6.070, *p* = 0.031, *η*^2^= 0.062)] was found, with increased dissimilarity (greater Euclidean distance) for phase 2 compared to phase 1 (Figure 4).

No other significant results were found. No significant results were found for O_2_Hb.

### Correlational Results

**Figure 5:**
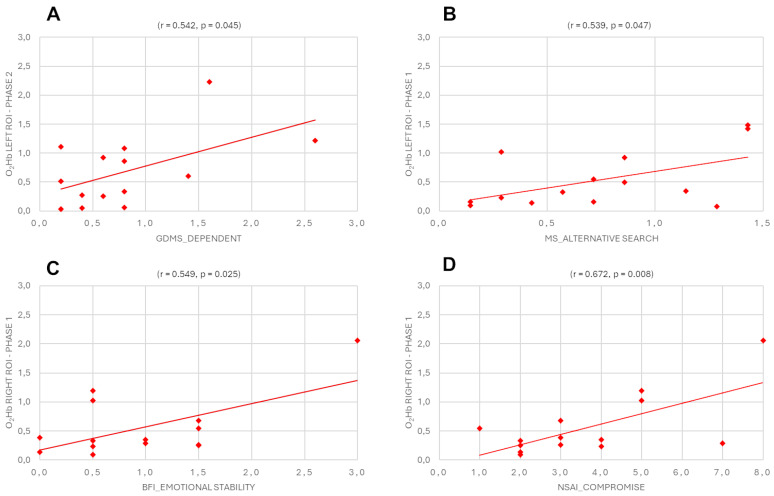
Pearson’s correlation between Eu_dis_ of O_2_Hb in the left and right hemispheres during Phase 1 and Phase 2 with Eu_dis_ of GDMS, MS, 10-item BFI, and NSAI. The scatterplots display (**A**) a positive correlation between GDMS-dependent style and the left ROI in Phase 2, (**B**) a positive correlation between MS alternative search and the left ROI in Phase 1, (**C**) a positive correlation between BFI emotional stability and the right ROI in Phase 1, and (**D**) a positive correlation between NSAI compromise and the right ROI in phase 1.

For O_2_Hb in the left and right hemispheres during Phase 1 and Phase 2 with GDMS, MS, and 10-item BFI, the results revealed a positive correlation between GDMS-dependent style and the left ROI in Phase 2 (r = 0.542, *p* = 0.045), a positive correlation between MS alternative search and the left ROI in Phase 1 (r = 0.539, *p* = 0.047), a positive correlation between BFI emotional stability and the right ROI in Phase 1 (r = 0.549, *p* = 0.025), and a positive correlation between NSAI compromise and the right ROI in Phase 1 (r = 0.672, *p* = 0.008) (Figure 5A–D).

**Figure 6:**
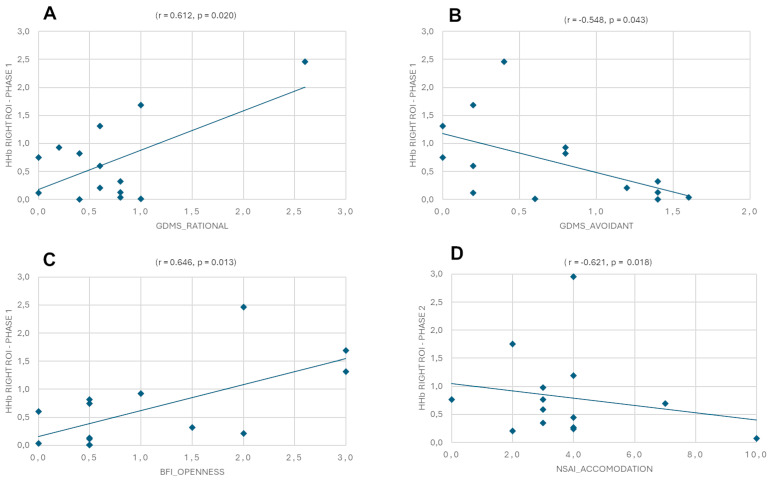
Pearson’s correlation between Eudis of HHb in the left and right hemispheres during Phase 1 and Phase 2 with Eudis of GDMS, MS, 10-item BFI, and NSAI. The scatterplots display (**A**) a positive correlation between the GDMS rational style and the right ROI in Phase 1, (**B**) a negative correlation between the GDMS avoidant style and the right ROI in Phase 1, (**C**) a positive correlation between BFI openness and the right ROI in Phase 1, and (**D**) a negative correlation between NSAI accommodation and the right ROI in Phase 2.

For HHb in the left and right hemispheres during Phase 1 and Phase 2 with GDMS, MS, and 10-item BFI, the results revealed a positive correlation between the GDMS rational style and the right ROI in Phase 1 (r = 0.612, *p* = 0.020), a negative correlation between the GDMS avoidant style and the right ROI in Phase 1 (r = −0.548, *p* = 0.043), a positive correlation between BFI openness and the right ROI in Phase 1 (r = 0.646, *p* = 0.013), and a negative correlation between NSAI accommodation and the right ROI in Phase 2 (r = −0.621, *p* = 0.018) (Figure 6A–D).

No other significant results were found.

## 4. Discussion

This study explores variations in hemodynamic coherence measures within couple dynamics when two decision-makers assume the roles of Pr and Pd, during a naturalistic decision-making interaction divided into two phases. Throughout this process, continuous data were collected from both the Pr and the Pd for O_2_Hb and HHb using fNIRS. Additionally, self-reported measures were used to assess individual differences (decision-making styles, maximization tendencies, personality traits, and negotiation skills).

First of all, the analysis identified significant effects at the dyadic level, demonstrating how the interaction phases between the Pr and the Pd influence the dyadic coherence. Specifically, Phase 2 (in which the Pd replies to the Pr and manages the communication exchange) revealed increased HHb dissimilarity in the PFC, suggesting greater inter-dyadic divergence during the Pd response.

As previously stated, only a study by Li et al. [12] has explored interpersonal neural synchronization between a Pr and a Pd, revealing that persuasive arguments generate stronger neural synchronization in key brain regions—namely, STG, SFG, and IFG—when compared to non-persuasive arguments. Analyses indicated that this neural synchronization occurs early in the persuasion process, signaling a pivotal moment when the Pd begins aligning with the Pr’s arguments. However, as most studies using fNIRS, the latter primarily focused on the exploration and interpretation of O_2_Hb.

Notably, Sato et al. [17] found that NIRS is a reliable tool for assessing hemodynamic signals arising from the PFC activation, demonstrating its comparability to BOLD signals obtained through fMRI, with lower levels of O_2_Hb and higher levels of HHb interpreted as markers of neural inhibition. HHb levels are closely associated with inhibitory mechanisms, as their increase is often interpreted as a marker of reduced neural activity or the suppression of specific brain regions during task engagement.

Indeed, the association of a lower level of HHb with the presence of O_2_Hb is grounded in earlier research showing that, under certain conditions, these two signal types can exhibit opposing responses during neural activation [18]. It is widely recognized that O_2_Hb signals are more sensitive than HHb signals to cerebral blood flow changes and exhibit a higher signal-to-noise ratio [19]. This might explain why most fNIRS studies have predominantly reported results based on O_2_Hb signals rather than HHb signals. Interestingly, the current study found increased dissimilarity in HHb (greater Eu_dis_) observed in Phase 2 compared to Phase 1, likely reflecting the shift in the interaction dynamics when the Pd takes on the role of speaker. Therefore, during Phase 2, the Pd’s response introduces a new perspective that may lead to a divergence in brain activity between the two individuals. Given that HHb levels are associated with neural inhibition [18] and their increase is often interpreted as a marker of reduced neural activity or of the suppression of specific brain regions during task engagement, this increased dissimilarity in HHb might reflect a change in the cerebral hemodynamic activity of the participants, particularly in regions associated with cognitive processing, attention, or emotional processing. The Pd’s active role in this phase likely introduces more complex cognitive and emotional responses, resulting in increased neural inhibition and disrupting the coherence initially established during Phase 1. Additionally, effective persuasion has been linked to the engagement of the Pr’s mentalizing system, including regions such as the dlPFC. This might suggest that the increased dissimilarity in hemodynamic activity during Phase 2 reflects the Pd’s greater efforts to adjust and integrate the persuasive message, as an attempt to reconcile their initial opinions with the ideas presented by the Pr, either aligning with the Pr’s viewpoint or debating against it [6,9,56].

Therefore, it could be argued that, during the persuasion process, there is more similarity in brain activity when the individual assuming the role of the Pr maintains control over the communicative dynamic and actively performs their role. Conversely, when the Pd speaks and responds and the Pr takes on a more passive role, the situation becomes more complex. This does not necessarily imply a breakdown in synergy or harmony within the dyad, but rather an increase in dissimilarity in the cognitive and neural processes involved. Notably, this result is further supported by the fact that the perceived efficacy of the persuasive interaction did not have any influence on the HHb levels. Regardless of whether the Pr employs a specific strategy or whether the outcome is perceived as effective or ineffective, when the Pd takes control of the interaction, dissimilarity is introduced.

Secondly, by taking into account the structural matching effect [30] and research on hemispheric specialization in processing persuasive messages—where the right hemisphere is more responsive to affective content and the left hemisphere shows a specialization for cognitive messages [6]—we conducted a correlational analysis to examine how dissimilarity in individual differences, including decision-making styles, maximization tendencies, personality traits, and negotiation skills (measured through the GDMS, MS, 10-item BFI, and NSAI), were associated with dissimilarities in the lateralization of hemodynamic activity during both phases of the shared decision-making process.

Specifically, results showed a positive correlation between dissimilarity in GDMS-dependent style and dissimilarity in O_2_Hb levels of the left hemisphere in Phase 2. Therefore, the greater the dissimilarity among the members in the use of a dependent style, the greater the dissimilarity in the left hemisphere activation (higher O_2_Hb) during Phase 2.

Usually, individuals with a dependent decision-making style are characterized by actively seeking suggestions and advice from others during the decision-making process [37]. Thus, the pattern we found might indicate an effort during this phase of the interaction to take a position on the shared decision without the possibility of directly asking someone else for advice, leading these individuals toward a preference for more cognitive processing (which is mediated by the left hemisphere) during the second phase of the persuasive process in shared decision making. In contrast, individuals with a less dependent style will probably rely more on affective processing of information, an approach that will lead to a decrease in left hemisphere activity.

On the contrary, as expected, the dissimilarity between the use of a rational decision-making style—characterized by a comprehensive search for information, evaluating all possible alternatives and their associated consequences—showed a positive correlation with increased dissimilarity in HHb levels in the right hemisphere during Phase 1. Indeed, the use of a rational decisional style might be associated with the suppression of affective processing (mediated by the right hemisphere) during the initial phase of the shared decision-making process, promoting a more logical, rational, and organized approach to information processing. On the other hand, individuals with a less rational style will rely more on affective processing, an approach that will increase right hemisphere activity.

An opposite correlational pattern was shown by dissimilarity in the use of an avoidant decision-making style—characterized by avoidance or delay in making decisions—which negatively correlated with dissimilarity in HHb levels in the right hemisphere in Phase 1. Notably, decision avoidance may be affected by negative emotions linked to anticipatory feelings such as fear and anxiety [57]. The existing evidence consistently suggests that some form of negative emotion, either generated by the choice itself [58,59,60] or arising from previous circumstances [61], typically precedes decision avoidance. Therefore, we might speculate that the increased dissimilarity in the use of an avoidant style related to a decreased dissimilarity in HHb levels (i.e., inhibition of the right hemisphere activity) reflects, during the initial phase, the occurrence of a negative emotion linked to the decision, mediated by the right hemisphere. On the contrary, individuals with a less avoidant style will rely on a preference for cognitive processing, thereby decreasing the activity of the right hemisphere.

Regarding personality traits—which are associated with individual tendencies in emotional or cognitive information processing in decision-making—results showed a positive correlation between dissimilarity in emotional stability and dissimilarity in O_2_Hb levels in the right hemisphere in Phase 1. Usually, those who are more emotionally stable generally show greater resilience, adaptability, and are linked to a preference for emotional information processing [62,63,64]; for this reason, they may exhibit a preference for affective processing during the initial phase of the persuasive process in shared decision-making that will produce an increased dissimilarity in O_2_Hb levels (greater right hemisphere activation). Such results might depend on the fact that those with low levels of emotional stability might prefer a more cognitive approach (leading to the inhibition of the right hemisphere).

On the other hand, individuals who showed dissimilarity in the openness trait—which, on the contrary, is linked to a preference for cognitive processing [63,64]—showed a positive correlation with the dissimilarity in HHb levels in the right hemisphere in Phase 1. Therefore, we might speculate that those with an openness trait who prefer cognitive processing present a relation with the inhibition of affective processing (mediated by the right hemisphere) during the early stages of the shared decision-making process, fostering a more logical, systematic, and rational approach to information processing. On the contrary, those with a low expression of the openness trait will probably rely more on emotional information processing, increasing the activation of the right hemisphere.

Finally, negotiation skills showed different patterns of correlation depending on O_2_Hb or HHb. Specifically, the compromise approach usually searches for a middle ground in resolving differences, involving understanding and integrating different perspectives, thus requiring emotional attunement. Therefore, the related increased dissimilarity in the level of O_2_Hb may reflect the fact that such an approach requires an emotional effort during the initial phase of the persuasive interaction to prepare for collaborative dialogue (i.e., increased activation in the right hemisphere). On the other hand, an opposite approach will rely more on a preference for cognitive processing, thus inhibiting the right hemisphere.

Instead, results showed a negative correlation between dissimilarity in accommodation and dissimilarity in HHb levels in the right hemisphere in Phase 2. Individuals who use an accommodation approach usually recognize that they were mistaken and are prepared to acknowledge the other person’s perspective or dislike conflict and want to restore harmony. Therefore, we might speculate that, in the second phase, individuals who score higher in accommodation may exhibit reduced affective processing or engagement of the right hemisphere, possibly implying that accommodating individuals are less likely to engage in affective evaluation. Instead, their focus might shift toward passivity or acceptance, reducing the need for active affective processing. On the contrary, less accommodating individuals will probably rely more on affective processing, thereby increasing the activity of the right hemisphere.

As a final result, analyses showed a positive correlation between dissimilarity in MS alternative search and dissimilarity in O_2_Hb levels in the left hemisphere in Phase 1. Individuals with a tendency toward alternative search usually invest significant time and resources in exploring all possible options; therefore, the related increased dissimilarity in O_2_Hb in the left hemisphere may reflect the cognitive effort (i.e., increased brain activity) during the initial phase of the persuasive interaction to systematically analyze information and evaluate alternatives. An opposite pattern will be shown by individuals with a low alternative search tendency, as they will probably rely more on a preference for affective processing of information which will decrease activity in the left hemisphere.

## 5. Conclusions

Overall, it can be suggested that, during the persuasion process, greater alignment in brain activity occurs when the individual acting as the Pr maintains control over the interaction and actively fulfils their role. In contrast, when the Pd takes the lead by speaking and responding, the dynamic becomes more intricate and challenging to manage. This does not necessarily indicate a disruption to the synergy or harmony but reflects an increase in cognitive and neural dissimilarity. Interestingly, this finding is further corroborated by the observation that the perceived effectiveness of the persuasive exchange had no impact on the HHb levels. Regardless of whether the Pr adopts a particular strategy or whether the interaction is seen as successful or unsuccessful, the Pd’s control introduces a degree of dissimilarity.

Furthermore, correlational results between dissimilarities in brain activity and individual differences revealed that Phase 1 of the interaction appears to be the one that produces the most dissimilarity when correlated with individual differences (i.e., decision-making styles, personality traits, and negotiation skills). The greater variety in emotional and cognitive responses observed during this phase may reflect the fact that people are actively presenting their arguments (Pr) and, on the other hand, elaborating on these arguments (Pd), a process that might have amplified differences between individuals.

From a methodological perspective, fNIRS is confirmed to be a useful tool for assessing and tracking brain function, due to its portability, resistance to noise, and because it is minimally invasive and highly adaptable across neurophysiological applications. For these reasons, it allows individuals to speak and interact naturally during recording, closely simulating everyday social situations and imposing minimal physical and psychological demands on participants. This makes it suitable for measuring brain activity in real-world and clinical settings [65]. Indeed, particularly in clinical settings, fNIRS functions not as a diagnostic tool but as an assistive device for monitoring functional brain activity and offering indirect indications of possible brain function abnormalities [21].

While this study offers the strength of introducing a dyadic perspective to the persuasion literature using an fNIRS hyperscanning approach and of achieving greater ecological validity by emphasizing the focus on interactive phases in which there is a realistic communicative exchange, there are several limitations to consider. A first limitation concerns the sample. Indeed, future studies should not only increase the sample size to improve the robustness and broaden the applicability of the results, but might also include a more diverse group of participants, incorporating, for example, professionals whose distinct characteristics or experiences might influence persuasive interactions in real-world decision-making situations to improve the generalizability of the findings to other populations or contexts. Additionally, this study did not analyze or consider potential gender-specific effects that might have influenced the persuasion process. Future research should examine the possible impact of this variable and further explore the persuasion process in both same-gender and mixed-gender dyads.

Secondly, while the PFC—a crucial cortical region in persuasive processes [5,10,11,12]—was the only area targeted by the fNIRS montage, future research should consider measuring other regions of the cerebral cortex, i.e., posterior areas such as the temporoparietal junction which is involved in understanding others’ perspectives and adjusting recommendations [7,66]. Additionally, future studies could also use a multimethodological hyperscanning approach by integrating simultaneous EEG, hemodynamic, and autonomic data collection to explore neuro- and psycho-physiological synchronization in dyads [67,68]; such an approach would provide a detailed understanding of the ongoing persuasive dynamics, encompassing both cognitive and emotional processes tied to the central and autonomic nervous systems.

Finally, a possible limitation could be the choice of the current method for measuring interbrain dissimilarity, as it is valid but not without its criticisms. For instance, to validate these findings, other analytical approaches could be considered to examine concurrent interbrain dynamics in both the Pr and the Pd, such as partial correlation, coherence analysis, cross-correlation, and canonical correlation analysis. These techniques have been used in prior research to investigate brain-to-brain coupling in hyperscanning studies [12,69].

## Figures and Tables

**Table 1:** Sample’s descriptive statistics.

		N	Mean Age
Dyad gender	Male–Male	4	26.25
	Female–Female	10	25.15
Total subjects	Male	8	26.25
	Female	20	25.15
Persuader	Male	4	29.25
	Female	10	26.21
Persuaded	Male	4	23.25
	Female	10	24.20

## Data Availability

The data presented in this study are available upon request from the corresponding author due to ethical reasons for sensitive personal data protection (requests will be evaluated according to the GDPR—Reg. UE 2016/679 and its ethical guidelines).
